# Digital Biomarkers in Psychiatric Research: Data Protection Qualifications in a Complex Ecosystem

**DOI:** 10.3389/fpsyt.2022.873392

**Published:** 2022-06-09

**Authors:** Andrea Parziale, Deborah Mascalzoni

**Affiliations:** Institute for Biomedicine, Eurac Research, Bolzano, Italy

**Keywords:** digital biomarkers, psychiatry, data protection and privacy, General Data Protection Regulation, controller, processor, law, ethics

## Abstract

Psychiatric research traditionally relies on subjective observation, which is time-consuming and labor-intensive. The widespread use of digital devices, such as smartphones and wearables, enables the collection and use of vast amounts of user-generated data as “digital biomarkers.” These tools may also support increased participation of psychiatric patients in research and, as a result, the production of research results that are meaningful to them. However, sharing mental health data and research results may expose patients to discrimination and stigma risks, thus discouraging participation. To earn and maintain participants' trust, the first essential requirement is to implement an appropriate data governance system with a clear and transparent allocation of data protection duties and responsibilities among the actors involved in the process. These include sponsors, investigators, operators of digital tools, as well as healthcare service providers and biobanks/databanks. While previous works have proposed practical solutions to this end, there is a lack of consideration of positive data protection law issues in the extant literature. To start filling this gap, this paper discusses the GDPR legal qualifications of controller, processor, and joint controllers in the complex ecosystem unfolded by the integration of digital biomarkers in psychiatric research, considering their implications and proposing some general practical recommendations.

## Introduction

In the EU, the authorization of medicinal products for marketing is heavily regulated (1). To be placed on the market, a medicinal product must be the object of a marketing authorization (MA) (2). Competent authorities release the MA for a product if the product's overall benefit-risk ratio is positive (3). This is based on an assessment of the safety and efficacy data gathered by the applicant pharmaceutical company in subsequent phases of clinical trials (3).

A key component of medicine development, clinical trials are also subject to stringent regulations, the aim of which is both to protect research participants and ensure that accurate and reliable data are generated (4). While Regulation (EU) No 536/2014 of the European Parliament and of the Council of 16 April 2014 on clinical trials on medicinal products for human use, and repealing Directive 2001/20/EC, which became applicable as of 31 January 2022 (5), promises to facilitate the conduct of multi-national trials across the EU, significant challenges are likely to remain. Typical clinical trial inefficiencies regard the recruitment and retention of research participants (6). For instance, (continued) participation in the research may prove burdensome, if not impossible, for individuals who reside far from the clinical trial site or have scheduling constraints (6). In turn, this may have a serious impact on data acquisition and, therefore, on the significance of clinical trial outcomes (6).

The digitalization of health research has the potential of addressing some of these challenges (6). The integration of digital devices and digital biomarkers into clinical trials is an important driver of health research digitalization (7). The term biomarker officially entered the medical vocabulary in 1988. That year, the US NIH working group adopted the following definition of biomarker: “a characteristic that is objectively measured and evaluated as an indicator of normal biological processes, pathogenic processes, or pharmacologic responses to a therapeutic intervention” (8). As such, biomarkers are integral to medical research and practice. Numerous traditional biomarkers, such as cholesterol levels, blood pressure, body temperature, and many other common measurements, are indeed well-accepted by both physicians and researchers (7). In clinical trials, such measurements help to objectively assess clinical statuses, therapeutic effects, and adverse events (9). Biomarkers are, therefore, crucial to understanding the mechanism of action of a medicine and supporting product development (10).

On the other hand, traditional biomarkers do not come without limitations. At the outset, the incorporation of biomarkers into medical research and practice is long and resource-intensive (7). In addition, biomarkers can be invasive and expensive (7). Also, traditional biomarkers often only offer a partial view of the patient's health status. This is because they can collect a limited number of measurements over time (“snapshot” problem) (7).

Digital biomarkers, i.e., “consumer-generated physiological and behavioral measures collected through connected digital tools” (11) or “objective, quantifiable, physiological, and behavioral measures collected by means of digital devices that are portable, wearable, implantable, or digestible” (12), arguably have the potential of addressing at least some of the traditional biomarkers' limitations (13, 14). Improvements in sensors, software, and algorithms, together with the increasingly widespread adoption of digital technologies in daily life, enable the gathering of key health-related data remotely, in non-invasive and seamless ways, blurring the boundaries between biomedical research and research participants' daily life (15, 16). In other terms, digital devices enable “continuous measurements outside the physical confines of the clinical environment” (17, 18). All this holds the potential of decreasing in-clinic assessment time and increasing statistical power, thus facilitating both participation in research and cost containment (7, 19). Examples of digital devices enabling the collection of biomarkers are numerous, ranging from wearable devices to sensors, to Internet of Things devices, and to smartphone applications (20–30).

Recognizing the potential benefit that may derive from biomedical research digitalization, regulators have been increasingly discussing policy strategies and regulatory tools to promote the integration of digital technologies into biomedical research (30). Generally, the twin aim of such initiatives is to create efficiencies and accelerate evidence generation while preserving the strength and reliability of randomized clinical trials. For instance, the Food and Drug Administration (FDA) and the European Medicines Agency (EMA) explicitly support the use of real-world data, also from wearables and biosensors, in regulatory decision-making (30).

In the same direction of facilitating the digitalization of health research, the FDA and EMA have been joined by Health Canada and the Japanese Pharmaceuticals and Medical Devices Agency to create data standards for digital health technologies (31). In particular, the FDA's Clinical Trials Transformation Initiative led to the adoption of general guidance on how digital technologies may be used to capture data in clinical trials (32).

These developments in digital and big data health research (33, 34) have unevenly impacted different medical specialties. Several medical areas, like oncology and neurology, have already progressed toward data-driven clinical decision-making based on a combination of subjective clinical assessment with digital, non-invasive biomarkers (35, 36). Conversely, as a discipline historically focused on subjective observation, psychiatry has only recently started to approach measurement-based care (35). However, there is an increasing recognition in the literature that data streams from digital sensors may improve modern psychiatric research and clinical practice, if combined with clinical observation and subjective self-reports (35). Also, integrating this complex information with modern computational and analytical methods holds the promise of advancing the field (35). Examples of psychiatric digital biomarkers include a smartphone application that detects early signs of treatment failure in chronic schizophrenia patients after discharge from hospital (37); realistic immersive simulations of daily situations to assess mild cognitive impairment (38); Machine Learning-based voice analysis to derive digital biomarkers of cognitive functioning in trauma survivors (39); dynamic tracking of change in the person's signatures as biomarkers in Alzheimer's disease, Parkinson's disease, frontotemporal dementia, depression, and schizophrenia (40); touch screens, keyboards, and microphones as biomarkers for motor control and speech in Alzheimer's patients, respectively (41). Likewise, performance in cognitive tasks on a smartphone application has been used to produce a validated Parkinson's disease score (42). In general, the psychiatric areas that seem most targeted by digital biomarkers include social anxiety, Parkinson's disease, autism, and mild cognitive impairment (43–50). In general, the social distancing and travel restriction measures adopted to counter the Covid-19 pandemic have arguably strengthened the case for conducting remote psychiatric clinical trials (51).

Despite its promising benefits, the increasing integration of digital biomarkers into psychiatric research does not come without risks. Digital biomarkers can capture an unprecedented amount of information about users, including fine-grained behavioral and physiological states (8). This poses several legal and ethical concerns (52) in an area already packed with them (53–57). Among these, privacy and data protection are not the least, especially for psychiatric patients (58). While patients are not necessarily hostile to data sharing, psychiatric patients are particularly wary of privacy issues (59–61). They are indeed aware that sharing “sensitive” data regarding their conditions and symptoms may negatively impact their daily life, also in terms of stigma and discrimination (10). These concerns potentially feed a feeling of distrust toward data sharing for research and, ultimately, discourage participation in research initiatives that are, nonetheless, essential to the development of much-needed medicinal products for the treatment of psychiatric conditions. Therefore, respecting privacy and data protection ethical and legal principles is not only a question of compliance. It is also key to establishing and maintaining a solid trust relationship among all the stakeholders involved in research, particularly between researcher and research participants (16, 58).

Despite this, privacy and data protection are often evoked (62) but rarely discussed in this domain. Two key contributions have started to partially fill this research gap, proposing some practical solutions in an attempt at reconciling the efficiencies promised by the digitalization of health research and practice with the respect of patients' fundamental rights and interests, including the rights to privacy and data protection (52, 63). These include the adoption of dynamic informed consent systems that allow participants to control the use of their data and change their preferences in an online environment; the implementation of transparent governance and oversight mechanisms; the certification of security measures; seeking Independent Review Board guidance; the conduct of data protection impact assessment; fostering the engagement of patients and/or their legal representatives.

What is still left out of the scope of the existing literature is a consideration of the positive data protection law in the context of digital biomarkers, starting from the preliminary issue of who is legally responsible for complying with data protection law and adopting these proposed solutions. Especially when digital devices and biomarkers are used, psychiatric research involves a complex set of different stakeholders, ranging from pharmaceutical companies to research teams, to the operators of the digital tools used to generate digital biomarkers, to automated algorithm-driven and AI-powered agents (64–71). Each of these human and non-human agents plays a distinct and often changing role in the research. In this complex and dynamic ecosystem, it may not always be clear how privacy and data protection duties and responsibilities are allocated.

Thus, this paper takes a step back *vis-à-vis* the extant literature on the relationship between data protection and psychiatric digital biomarkers. In particular, it aims to map the issues related to the GDPR qualifications of the actors involved and their practical impact on the integration of digital biomarkers into psychiatric biomedical research initiatives.

To this end, this paper is structured as follows. First, it provides a summary of the principles and main legal qualifications outlined in Regulation (EU) 2016/679 of the European Parliament and of the Council of 27 April 2016 on the protection of natural persons with regard to the processing of personal data and on the free movement of such data, and repealing Directive 95/46/EC (General Data Protection Regulation, GDPR). Secondly, the main stakeholders involved in digital biomarker-powered psychiatric research are identified. Thirdly, the GDPR legal qualifications are applied to each stakeholder, with a view to identifying their respective data protection duties and responsibilities. Finally, the conclusions summarize the preceding analysis and provide some general practical advice. This clarification of data protection legal responsibilities will help companies and researchers in their efforts to expedite psychiatric research in a way that is compliant with data protection laws and principles and effectively ensures and maintains participants' trust in the research.

## GDPR Summary

The integration of digital biomarkers into psychiatric research implies the production and sharing of massive amounts of personal data. In the EU, the use of data in this context falls under the scope of the GDPR. The GDPR is based on several principles that govern the processing of personal data (Article 5). First, the processing of personal data must be lawful, fair, and transparent. Secondly, data must be “collected for specified, explicit and legitimate purposes and not further processed in a way incompatible with those purposes” (so-called purpose limitation principle). Thirdly, the data must be limited to what is strictly needed to pursue such purposes (data minimization principle). Likewise, data can be kept only for the time that is necessary to pursue the purposes of the processing (storage limitation principle). Finally, under Article 5(2) of the GDPR, data must be accurate and used securely. The data controller must ensure, and be able to demonstrate, compliance with all these principles (accountability principle).

The use of health-related and genetic data is subject to specific rules. Health data are indeed “special categories of data” (formerly known as sensitive data). These data generally cannot be processed [Article 9(1) GDPR]. This is unless one of the exceptions listed in Article 9(2) applies. These include circumstances when the data subject explicitly consented to the use of their data; the use of data is necessary for the performance of legitimate activities of not-for-profit bodies, subject to appropriate safeguards; the data subject makes the data public; the processing of data is necessary for reasons of substantial public interest, including public health, as stated in an EU or Member State law that provides for appropriate safeguards; the use of the data is necessary for research, as set out in EU or Member State law and provided that appropriate safeguards are adopted. It is the data controller that must assess whether one of these exceptions applies and ensure that appropriate safeguards are in place to protect the data subject's fundamental rights.

The GDPR also provides data subjects with several rights. In particular, the data subject has the right to information on the processing of their personal data and their rights (Article 13 GDPR). The data subject has also the right to access the personal data that the controller stores on them (Article 15 GDPR); the right to have inaccurate personal data rectified (Article 16 GDPR); and the right to the erasure of personal data, e.g., if the data subject withdraws their consent, and no other lawful basis for the processing applies (Article 17 GDPR).

The use of data for scientific research purposes is subject to a special regime. In this case, the GDPR foresees derogations from several data protection requirements. For instance, personal data may be stored for longer than necessary and used for a research purpose not originally specified (Article 5(1)(c) and (e) GDPR). The GDPR also foresees derogations from several data subjects' rights, including the right to access, rectification, erasure, and object. These derogations to the data subject's rights are permitted if the exercise of one of these rights is likely to render impossible or seriously impair the achievement of the objectives of the research [Article 89(2) GDPR].

Nevertheless, the research exemptions may only apply if the data controller adopts safeguards to protect “the rights and freedoms of the data subject” (Article 89 GDPR). Such safeguards must be “appropriate,” “in accordance with the GDPR,” and “ensure that technical and organizational measures are in place in particular in order to ensure respect for the principle of data minimization.” While Article 89 GDPR does not elaborate further on this, the EDPB provided some indications on potential safeguards in the research context (72, 73). Also, additional guidance may be found in soft law and legal instruments that regulate the use of health data for research (74).

Finally, the GDPR regulates cross-border data transfers. Outside the European Economic Area (EEA), data transfers are permitted if the recipient country is covered by an adequacy decision by the European Commission ensuring that that country has a level of protection equivalent to that ensured by the GDPR. In lack of an adequacy decision, transfers are permitted if safeguards are in place, such as standard contractual clauses, binding corporate rules, approved codes of conduct, or approved certification mechanisms (Article 46 GDPR). In this case, the third country must provide a level of protection of data subjects that is equivalent to that ensured by the GDPR. To assess this, the data exporter should map the transfers; identify the safeguard to be used for the data transfer; assess the laws of the third country, also with the assistance of experts in the laws of the third country, considering all data protection laws and actual practices in the third country, the power of the authorities to access personal data for surveillance, and the existence of an effective right to judicial redress; consider other supplementary contractual, technical, or organizational measures to elevate the level of protection to the one ensured by the GDPR (75).

In lack of an adequacy decision and Article 46 safeguards, data transfers are permitted if one of the exceptions set out in Article 49 of the GDPR applies. These include the explicit consent of the data subject, the protection of the vital interests of the data subject, and important reasons of public interest, such as the fight against a cross-border health threat (e.g., a pandemic) (76). However, these exceptions are to be construed restrictively (76) and, therefore, cannot apply to research generally.

## GDPR Qualifications: Controller and Processor

The GDPR foresees several legal qualifications. These include a controller that “determines the purposes and means of the processing of personal data” and a processor that “processes personal data on behalf of the controller” (Article 4 GDPR). Also, two or more controllers that “jointly determine the purposes and means of processing” qualify as joint controllers under Article 26 of the GDPR.

As anticipated in the introduction, it may not always be clear how these qualifications are allocated in the context of psychiatric research that embeds digital biomarkers. Indeed, the integration of digital biomarkers in psychiatric research operates in a complex ecosystem in which several different actors play distinct roles. Although this may change from case to case, the involved actors typically include the sponsor of the clinical trial, i.e., the pharmaceutical company sponsoring the research on an investigational medicinal product in view of obtaining a MA; the team of researchers that conduct the clinical trials; and the operator of the digital tool (e.g., a wearable or a smartphone app) that is used for the production of a digital biomarker. Other actors that may be involved in this ecosystem include healthcare service providers and biobanks. Indeed, key insights into the effects of an investigational product may be obtained by combining digital biomarkers with (electronic) medical health records and human biosamples (77–79), respectively.

Additional guidance on these definitions is in the EDPB Guidelines 07/2020 on the concepts of controller and processor in the GDPR (hereinafter also referred to as “EDPB Guidelines”) (80). The EDPB Guidelines clarify that the concepts of controller and processor are functional and must be determined based on a factual analysis. This is irrespective of the terms and conditions utilized in contractual arrangements. In other words, the controller decides on the purposes and essential elements of the means of the processing, such as the data that will be used, the storage period for the data, and who will have access to such data. In contrast, non-essential aspects concerning the means of the processing may be devolved to a processor.

The EDPB Guidelines also clarify that the processor is: separate from the controller, i.e., the processor must not belong to the organization of the controller (which rules out the employees of the controller); and processes personal data on behalf of the controller, i.e., the processor operates in the interest of the controller. In other terms, the processor must comply with the instructions of the controller and does not have any interest of their own.

## Digital Biomarkers in Psychiatric Research: The Sponsor- Investigator Relationship

The summary of the GDPR sections and qualifications and EDPB guidance outlined above enables an orderly discussion of how these qualifications are distributed in the complex ecosystem of psychiatric research that makes use of digital biomarkers.

In such a context, a clinical trial sponsor likely qualifies as a controller. This is because they primarily determine the purpose and means of the processing by drafting the research protocol. In particular, the purpose is scientific research, with a view to testing the safety and efficacy of an investigational product for the treatment of a psychiatric condition to obtain an MA.

As a controller, the sponsor must ensure, and be able to demonstrate, compliance with the data protection principles set out in Article 5 of the GDPR. In particular, the sponsor must ensure that the processing is based on a valid legal basis under both Articles 6 and 9 of the GDPR, since special categories of data are processed. Irrespective of the selected legal basis, the controller must provide the data subjects with information on the processing, including, but not limited to, the identity of the controller, the contact details of the data protection officer, the purposes and the legal basis of the processing, the recipient of the personal data, whether the controller intends to transfer data to a third country or an international organization, the storage period, the data subjects' rights, the right to lodge a complaint before the competent supervisory authority, and the existence of automated decision-making, including profiling, and its underlying logic. This latter information is required if AI and Machine Learning tools are used in the clinical trial to automatically gather and elaborate the personal data of the research participants.

Relatedly, the sponsor must enable the effective exercise of the data subjects' rights [Article 14(2) GDPR]. Thirdly, the sponsor must implement appropriate technical and organizational measures to comply with data protection legislation (Article 24 GDPR). In particular, they must implement appropriate technical and organizational measures (such as pseudonymization) to comply with the data protection principles effectively (such as data minimization) and integrate the necessary safeguards to comply with the requirements of the GDPR and safeguard the rights of the data subjects [Article 25(1) GDPR: privacy by design]. Furthermore, they must adopt appropriate technical and organizational measures to ensure that, by default, only the personal data necessary for the processing are used [Article 25(2) GDPR: privacy by default]. Moreover, the sponsor is likely required to conduct a Data Protection Impact Assessment (DPIA). This is because the use of “sensitive” data, such as health-related and genetic data likely entails risks for the data subjects. Finally, the sponsor must ensure that data transfers outside the EEA are based on an adequacy decision, appropriate safeguards, or an exception under Article 49 of the GDPR (which is, anyway, unlikely to apply to research).

Failure to comply with one of the GDPR obligations may result in the sponsor being targeted by compensation claims filed by data subjects [Article 82(2) GDPR]. This is unless the sponsor proves that they were in no way responsible for the event that caused the damage [Article 82(3) GDPR]. Administrative fines may also be enforced by the competent supervisory authority up to 10 million euros, or 2% of the global turnover if the infringer is a company (Article 83 GDPR).

In any event, in the development of psychiatric medicines using digital biomarkers, the sponsor is likely to collaborate with other, separate organizations. These may include a team of researchers (hereinafter also referred to as investigator) and the operator of the digital tool that is used to generate the digital biomarkers. Other organizations may also be involved, such as healthcare service providers and biobanks or databanks, for data linkage purposes.

In this connection, situations of joint controllership or controller-processor relationships may arise. For joint controllership to arise, two or more parties must determine together the purposes and means of the processing (Article 26 GDPR). The EDPB Guidelines clarify that this must be assessed with a substantive and functional approach. In particular, joint controllers jointly participate in the determination of the purposes and essential elements of the means of the processing, irrespective of the terms of contractual arrangements.

The EDPB Guidelines also clarify that such joint participation may stem either from a “common decision” or “converging decisions.” To determine if converging decisions are made, “an important criterion is that the processing would not be possible without both parties' participation in the sense that the processing by each party is inseparable, i.e., inextricably linked. The joint participation needs to include the determination of purposes on the one hand and the determination of means on the other hand.”

The EDPB Guidelines outline an example that regards collaborative research projects. In this example, several institutes decide to join a collaborative research project, send personal data to a common platform, and use such data for research. The EDPB Guidelines state that they are all joint controllers. This is because they have co-determined the purposes (i.e., the joint research) and means (i.e., the use of the platform) of the processing. However, if decontextualized, this example provided by the EDPB may be misleading. For joint controllership to arise, it is not sufficient for the partners to undersign a joint research project. Rather, the relevant test is to assess the actual influence on the (co-)determination of the purposes and means of the processing.

In the scientific research context, one way of assessing this is to identify who actually participated in the drafting of the research protocol. This is confirmed by another example provided by the EDPB Guidelines, which specifically regards the qualifications of the sponsor and the investigator of a clinical trial. In particular, the example considers the case of an investigator and a sponsor that decide to start a clinical trial and collaborate on the drafting of the research protocol. The research protocol sets out, among other things, the purpose and the methodology of the study, as well as the types and amount of data to be collected. Under these circumstances, according to the EDPB, the investigator and the sponsor can be considered joint controllers. This is because, for this clinical trial, they determine together the purpose and the essential aspects of the means of the processing. Conversely, if the clinical trial protocol is drafted only by the sponsor and the investigator just accepts it, the sponsor is the controller, and the investigator is the processor for the clinical trial.

These different qualifications entail the application of different sets of rules under the GDPR. When joint controllership arises, the sponsor and the investigator must allocate their respective GDPR responsibilities [Article 26(1) GDPR], particularly regarding the rights of the data subject and the information obligations outlined in Articles 13 and 14. However, the Court of Justice of the EU (CJEU) held, and the EDPB Guidelines confirmed, that “joint responsibility does not necessarily imply equal responsibility of the various operators involved in the processing of personal data” [Court of Justice of the EU (CJEU), judgment of 5 June 2018, C-210/16, *Wirtschaftsakademie*, paragraph 43]. The EDPB Guidelines further clarify that the allocation of the respective responsibilities should consider who is best placed to comply with the relevant GDPR obligations and, particularly, to ensure the data subject's rights (80). In line with the accountability principle, the Guidelines recommend that the internal analysis underlying the allocation of the different responsibilities should be properly documented.

To this end, the joint controllers must conclude “an arrangement between them” and the “essence” of such an agreement must be made available to the data subjects (Article 26 GDPR). The EDPB Guidelines recommend that the arrangement should be a legally binding contract (80).

Thus, joint controllers have a certain margin of discretion in the allocation of their respective responsibilities and can adjust them to their actual role in the different stages of the data processing operation. However, irrespective of the joint controllership arrangement's terms and conditions, the data subjects can exercise their GDPR rights against each controller [Article 26(3) GDPR]. As a result, data subjects can always ask either the sponsor or the operator for consent withdrawal or data erasure.

Likewise, joint controllers are jointly and severally liable for the entire damage caused by a GDPR infringement (Article 82 GDPR). The controller that pays full compensation has redress toward the other joint controller. In a redress action, the terms of the joint controllership agreement may become relevant to the apportionment of liability between the joint controllers. In any event, a controller is exempt from liability if they are not “in any way responsible for the event causing the damage” [Article 82(3) GDPR].

Conversely, there is no indication in the GDPR that a supervisory authority may impose joint and several liability on joint controllers for administrative fines. The CJEU held that the “level of responsibility of each of [the joint controllers] must be assessed with regard to all the relevant circumstances of the particular case” (CJEU, judgment of 5 June 2018, C-210/16 *Wirtschaftsakademie*, paragraph 43). Thus, an organization “cannot be considered to be a controller in the context of operations that precede or are subsequent in the overall chain of processing” on which it has no influence (CJEU, judgment of 29 July 2019, C-40/17, *Fashion ID*, paragraph 74).

Different rules apply if a controller-processor relationship arises between the sponsor and the investigator. In such circumstances, the controller must use “only processors providing sufficient guarantees to implement appropriate technical and organizational measures” [Article 28(1) GDPR]. To this end, the sponsor should take into account the technical expertise, reliability, and resources of the processor (Recital 81 GDPR). Also, the processing by a processor must be governed by a written contract (also in electronic form) concluded by the controller and processor [Article 28(3) GDPR]. This contract must provide, among other things, that the processor complies with the “documented instructions from the controller, including with regard to transfers of personal data to a third country” [Article 28(3) GDPR].

In turn, the processor must fulfill the obligations set out in Article 28 of the GDPR. In particular, the processor is bound to the documented instructions of the controller. The processor must also subject the persons authorized to process data to confidentiality and implement technical and organizational measures to ensure data security. Moreover, the processor must support the controller in the fulfillment of the obligations of the controller toward the data subjects. Furthermore, the processor is required to delete or return, as determined by the controller, all data to the controller upon the termination of their services. Finally, the processor cannot resort to a “sub-processor” without the authorization of the controller.

In sum, the controller exerts strict control over the processor. While this ensures that the processor does not misuse the data, the sponsor retains primary responsibility for compliance with the GDPR, including safeguards and technical and organizational measures (e.g., Articles 5, 24, and 25 GDPR). In contrast, the processor is only liable for infringing either the few GDPR direct processor obligations or the documented instructions of the controller (Article 28 GDPR).

Likewise, when qualifying as a processor, the investigator is liable for the damages caused by the processing only if they violate one of the processor-specific obligations outlined by the GDPR or the controller's lawful instructions (Article 82(2) GDPR). However, like the controller, the processor is exempt from liability if it is not “in any way responsible for the event giving rise to the damage” [Article 82(3) GDPR]. Finally, the processor is subject to administrative fines in case of non-compliance (Article 83 GDPR).

## Digital Biomarkers in Psychiatric Research: The Role of the Digital Tool Operator, Electronic Medical Record Provider, and Biobank / Databank

Ultimately, the “research-protocol-drafting” test outlined above draws a clear distinction between the cases in which the investigator and the sponsor are joint controllers and the cases in which the investigator qualifies as a processor. However, such a test does not really work when it comes to another key actor of the ecosystem considered, i.e., the operator of the digital tool used to generate the digital biomarkers. The operator arguably qualifies as a (joint) controller, even when the operator does not participate in the drafting of the scientific research protocol.

In this case, the operator would qualify as a processor if the sponsor pursued their own research purpose and the operator shared data with the sponsor at the mere request of this latter. In this case, the operator would not have any influence in the determination of the purposes and means of the processing and would therefore act in the exclusive interest of the sponsor.

However, key data protection principles, such as lawfulness, purpose and storage limitation, and data minimization, prevent the operator from sharing personal data with third parties in an unrestricted way, especially when it comes to “sensitive” data, such as health-related data. Under these circumstances, the operator does not act in the mere interest of the sponsor. Indeed, it is the operator that ultimately decides whether or not to share the patient's personal data with the sponsor. In fact, without this decision, the processing would not take place altogether. This applies also when the operator does not participate in the design of the research protocol and just accepts it as proposed by the clinical trial sponsor (and investigator).

Against this backdrop, the concept of converging decisions outlined in the EDPB Guidelines (80) has the potential of clarifying this gray area between controllership and processorship. This is because both the sponsor (and investigator) access request and the operator' decision to share the data are indispensable for the processing to take place.

The same likely applies to healthcare service providers and biobanks / databanks from which the sponsor (and investigator) may want to obtain access to health-related data (including medical records), genetic data, and human biosamples. Indeed, data protection principles require controlled-access models for the sharing of health-related and genetic data and samples (74). This makes both the access request by the sponsor (or the investigator) and the decision of the data provider to share data “inextricably linked” and indispensable for the processing to take place. Thus, data providers such as medical record providers and biobanks / databanks are unlikely to qualify as mere processors and more likely to be considered as joint controllers, based on converging decisions. The same applies to databanks or platforms established by clinical trial sponsors to share clinical trial data with external researchers conducting their own independent research.

## Conclusions

In the complex and dynamic ecosystem unfold by the integration of digital biomarkers into psychiatric research, the appropriate management of the relationships among the involved actors is key to ensuring compliance and maintaining participants' trust in the research.

The discussion above first indicates that the allocation of the GDPR qualifications is not a formal exercise. This is in a 2-fold sense. On one hand, the actors involved in data processing cannot arbitrarily qualify themselves as controllers or processors when negotiating their contractual relationships. Indeed, what is specified in contractual arrangements may be indicative of these qualifications. However, what is decisive is the actual influence exerted by each actor in the determination of the purposes and means of the processing. On the other hand, being a controller or processor is not just a legalistic matter of labels. Each qualification implies distinct duties and responsibilities.

In the context considered in this paper, the relationships between the stakeholders may give rise either to joint controllership situations or controller-processor relationships. These qualifications entail distinct legal implications. On one hand, joint controllers enjoy relative discretion to distribute their duties and responsibilities among themselves, based on their involvement in the different segments of the processing. On the other hand, in a controller-processor relationship, the former has stricter control over the latter but retains primary liability for GDPR compliance. This is unless the processor fails to comply with the documented instructions of the controller.

Against this backdrop, joint controllership seems a likely outcome of the data sharing entailed by the integration of digital biomarkers in psychiatric research. In particular, even when the operator of the digital tool used to generate the digital biomarkers adheres to a research protocol designed by the sponsor (and the investigator), the operator likely qualifies as a joint controller based on converging decisions. Thus, the sponsor and the operator must conclude an agreement to clearly allocate their duties and responsibilities and share this with the research participants. This allocation should consider who is best placed to fulfill the respective duties, and the analysis underlying this allocation should be properly documented. The joint controllership agreement should also regulate the rights of the research participants.

Another critical aspect that should be addressed in joint controllership and controller-processor agreements is the parties' civil (or tort) liability for damages caused by the use of personal data. In this respect, each controller and processor are, in principle, liable for the entire damage caused by the processing, with redress rights toward the other parties involved. To clarify their respective civil liability exposure, the controller and processors should consider including clauses that apportion the damages due by each party and/or indemnification clauses. Whether these latter may also cover administrative fines depends on the applicable national law.

## Author Contributions

AP conceptualized the paper and developed the first draft. DM provided important intellectual input and constructive comments on the first draft. All authors reviewed and approved the final draft.

## Funding

At the time during which the work was undertaken, AP and DM were funded by the CHRIS 2D project under the European Regional Development Fund (EFRE).

## Conflict of Interest

The authors declare that the research was conducted in the absence of any commercial or financial relationships that could be construed as a potential conflict of interest.

## Publisher's Note

All claims expressed in this article are solely those of the authors and do not necessarily represent those of their affiliated organizations, or those of the publisher, the editors and the reviewers. Any product that may be evaluated in this article, or claim that may be made by its manufacturer, is not guaranteed or endorsed by the publisher.
